# Chemical Composition and Insecticidal Activity of Essential Oils from *Origanum floribundum* and *Eucalyptus citriodora* Against the Louse *Bovicola limbatus*

**DOI:** 10.3390/molecules30194001

**Published:** 2025-10-06

**Authors:** Nassima Chikhi-Chorfi, Fairouz Haddadj, Baya Djellout, Safia Zenia, Mohamed Hazzit, Faiza Marniche, Amel Milla, Amina Smai

**Affiliations:** 1 Laboratory of Reaserch “Health and Animal Productions”, Higher National Veterinary School, Road Issad Abes, Oued Smar, Algiers 16000, Algeria; b.djellout@ensv.dz (B.D.); s.zenia@ensv.dz (S.Z.); a.milla@ensv.dz (A.M.); 2 Preclinical Department, Higher National Veterinary School, Road Issad Abes, Oued Smar, Algiers 16000, Algeria; f.haddadj@ensv.dz (F.H.); f.marniche@ensv.dz (F.M.); a.smai@ensv.dz (A.S.); 3 Laboratory for Fundamental Research in Operational Research, Combinatorics and Econometrics (IFORCE), Faculty of Mathematics, University of Sciences and Technology, Houari Boumediene, B.P.32, Algiers 16111, Algeria; 4 Department of Food Technology and Human Nutrition, National High School of Agronomy, Algiers 16004, Algeria; mohamed.hazzit@edu.ensa.dz

**Keywords:** essential oil, insecticidal activity, *O. floribundum*, *E. citriodora*, *B. limbatus*

## Abstract

Background: Essential oils, obtained from plants, are an alternative for controlling ectoparasites, particularly lice, mites and ticks, due to the problems posed by chemical insecticides, such as insect resistance, environmental impacts and concerns related to human and animal health. This study aims to investigate and compare the insecticidal activity of essential oils from *Origanum floribundum* and *Eucalyptus citriodora* against the louse *Bovicola limbatus*. Methods: The chemical composition of the two oils obtained by hydrodistillation was determined by gas chromatography coupled with mass spectrometry (GC-MS) and a flame ionisation detector (FID-MS). To determine insecticidal activity, the essential oils were tested at different concentrations (0.05–0.8 µL/mL), with mortality recorded after 15 min, 30 min, 1 h, 2 h and 4 h of exposure. Results: A corrected mortality rate of 100% was achieved for concentrations of oregano and eucalyptus essential oils of 0.8 µL/mL and 0.4 µL/mL, respectively. The LC_50_ values were 0.11 and 0.10 µL/mL for oregano and eucalyptus, respectively, after 2 h of treatment. The LC_90_ values observed are 0.31 and 0.24 µL/mL for oregano and eucalyptus, respectively. Conclusion: Both essential oils have similar and promising insecticidal potential and could be an alternative to chemical insecticides in a control strategy that is more respectful of human and animal health and the environment.

## 1. Introduction

In Algeria, goat farming is present in all areas. In the north, it is confined to mountainous areas, but the bulk of the population is distributed across steppe and sub-desert areas [1,2]. Goats play a crucial role in human societies, particularly in marginal and rural areas. They provide essential resources such as milk, meat, fibre (including cashmere and mohair) and manure, contributing to food security, income generation and sustainable agricultural practices. Their ability to adapt to a variety of environments makes them invaluable assets for supporting livelihoods around the world [1,3,4]. Generally, this type of farming is carried out extensively [1,3,5], which increases the risk of infestation by ectoparasites, particularly lice.

Mallophagous lice of the genus *Bovicola* are a major problem for small ruminant farmers around the world [6,7]. The negative effects of infestations by these lice have been reported in the literature [6,8]. These ectoparasites threaten animal welfare and the economic profitability of many ruminant farming systems around the world [9]. Lice are mainly present in farms where hygiene, feeding and storage conditions do not meet requirements [10]. Currently, there is little research on lice infestation in small ruminants [11], and very little is known about the effects of lice infestation in goats [6].

Today, ectoparasite infestations in livestock are mainly controlled using synthetic pesticides. However, excessive use of these pesticides has led to the growing and worrying development of resistance to chemical pesticides in insect populations [12]. Often, to overcome this resistance, it is necessary to use higher doses of pesticides and more frequent applications, which exacerbates environmental contamination, increases economic costs and heightens risks to human health [13,14].

To address the dangers associated with the increased use of chemical pesticides, there is now growing interest in the study and evaluation of botanical insecticides for pest control [15]. These botanical insecticides could be a possible natural alternative to synthetic pesticides and help reduce the harmful impact of chemicals on the environment and humanity [12].

*O. floribundum* and *E. citriodora* are plant species belonging to the Lamiaceae and Myrtaceae families, respectively. The essential oils of both plants are characterised by a wide range of biological activities, including antimicrobial, antifungal, antioxidant and insecticidal properties [16,17,18,19,20,21,22]. In Algeria, the genus Origanum is represented by two species that grow wild: *O. vulgare* L. subsp. *glandulosum* (DESF.) Ietswaart, and the rare endemic species *O. floribundum* Munby [17,23]. In Algeria, phytochemical research has mainly focused on the common species, with studies on *O. floribundum* remaining rare [17].

The objectives of this study are to determine the chemical compositions of the essential oils of *E. citriodora* originating from Algeria and *O. floribundum*, a rare endemic species that grows wild in north-central Algeria [24], and to evaluate and compare their insecticidal effect on the mallophagous louse *B. limbatus*.

## 2. Results

### 2.1. Yield and Chemical Composition

Table 1 lists the yields, chemical classes and GC data for components identified in *O. floribundum* and *E. citriodora* essential oils. Chromatograms, GC-MS analysis and mass spectra of the main components are provided as Supplementary Materials Files.

Chromatographic analysis led to the identification of a total of 38 compounds in the essential oil of *O. floribundum* and 27 in that of *E. citriodora*. *p*-Cymene (10.78%), γ-terpinene (18.91%) and carvacrol (54.63%) were the most prominent constituents of *O. floribundum* EO, constituting the bulk of this oil. Isopulegol (18.9%), citronellol (10.94%) and citronellal (58.01%) were the main components of *E. citriodora* EO, representing 82.85% of the total oil. Both essential oils are dominated by oxygenated monoterpenes (60.37% vs. 93.38% for *O. floribundum* and *E. citriodora*, respectively).

### 2.2. Morphological Identification

**Figure 1:**
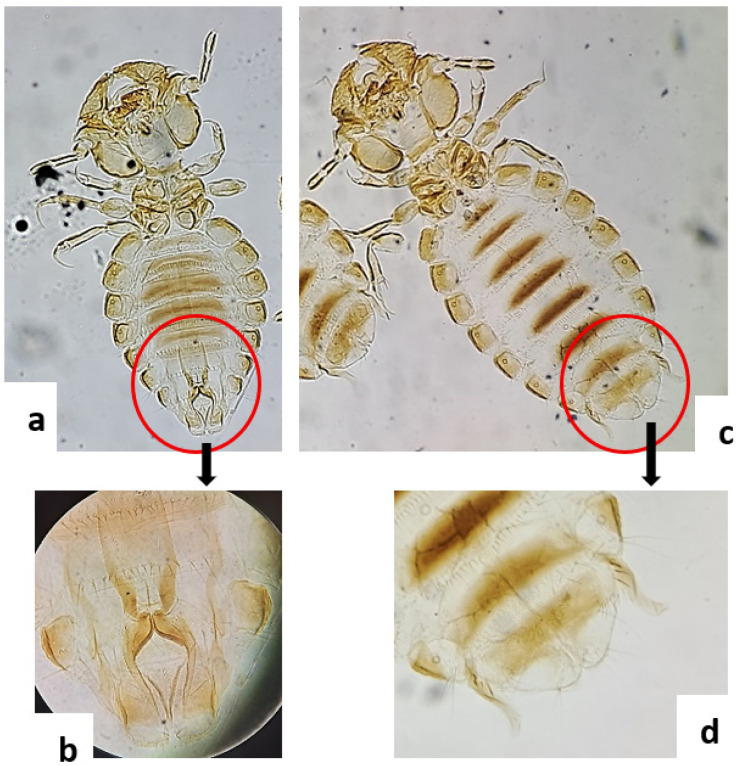
*Bovicola limbatus* (Mallophaga) viewed under Leica DM500 microscope. (**a**) male; (**b**): male genitalia; (**c**): female; (**d**): female genitalia.

Microscopic examination of the lice samples collected from the goat herd revealed the presence of *B. limbatus*, a chewing louse specific to goats. Based on external morphological characteristics, particularly at the abdominal extremity, male individuals (Figure 1a,b) and female individuals (Figure 1c,d) were successfully distinguished.

### 2.3. Contact Toxicity

**Figure 2:**
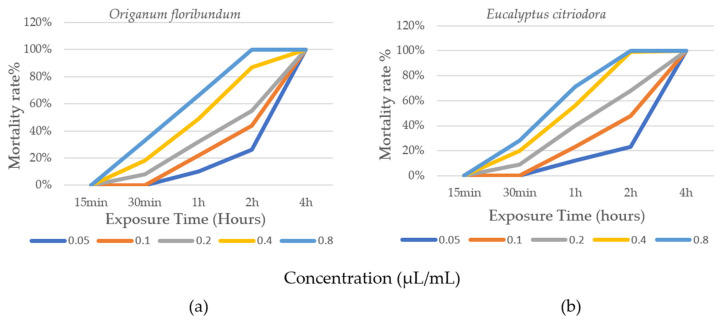
Evolution of the biocidal effect of EO according to concentration and exposure time. (**a**) *Origanum floribundum;* (**b**) *Eucalyptus citriodora*.

Figure 2a,b illustrate the progression of insecticidal activity of essential oils from *O. floribundum* and *E. citriodora* applied to *B. limbatus* lice, depending on the concentration and exposure time of the insects. The corrected average mortality rates based on concentration and treatment duration are presented in Table 2.

The results obtained show a progressive increase in the corrected mortality rate of *B. limbatus* lice for the concentrations (0.1 μL/mL; 0.2 μL/mL; 0.4 μL/mL; and 0.8 μL/mL) applied throughout the treatment period (Figure 2a,b; Table 2). The 0.05 μL/mL concentration had only a marginal effect during the first hours (1 h and 2 h) compared to the other concentrations, particularly C_4_ and C_5_. During the first 15 min of exposure, no mortality was recorded at any concentration of either essential oil (Table 2). After 30 min of exposure at 0.8 μL/mL, *O. floribundum* and *E. citriodora* essential oils showed toxicity with corrected mortality rates of 33% and 28%, respectively. After 1 h of exposure at the same concentration, corrected mortality rates reached 66% for oregano and 71% for eucalyptus. After 2 h, 100% corrected mortality was achieved at 0.8 μL/mL for oregano, and at both 0.4 μL/mL and 0.8 μL/mL for eucalyptus. For the same treatment duration, corrected mortality rates at 0.05 μL/mL, 0.1 μL/mL, and 0.2 μL/mL were 26%, 44%, and 55% for oregano, and 23%, 48%, and 68% for eucalyptus, respectively. After 4 h of exposure, all five concentrations resulted in 100% mortality of the lice. The absence of mortality in the control group confirms the biocidal efficacy of the tested EO.

**Figure 3:**
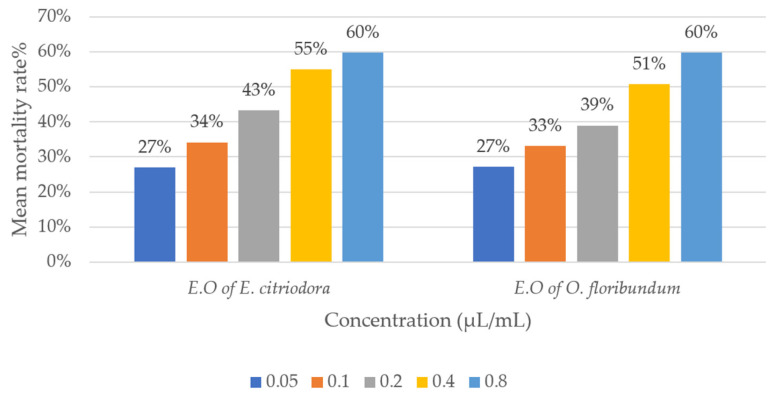
Comparison of the corrected mortality rates of *Bovicola limbatus* lice.

Statistical analysis of the corrected mortality rates of lice treated with *O. floribundum* and *E. citriodora* essential oils revealed significant variations depending on the studied factors (Table 3). ANOVA showed a highly significant effect of essential oil concentration and exposure duration on mortality rates (*p* < 0.0001). In contrast, the type of extract had no significant effect (*p* = 0.609). These findings indicate a dose- and time-dependent relationship in the insecticidal effect of the oils on *B. limbatus*. The increase in corrected mortality rate is proportional to both the concentration and the duration of treatment. However, the nature of the extract did not show a significant effect, suggesting that the insecticidal activity of *O. floribundum* and *E. citriodora* essential oils does not differ significantly (Figure 3).

### 2.4. Toxicity Parameters of the Essential Oils of O. floribundum and E. citriodora

The lethal concentration values CL_50_ (lethal concentration to kill 50% of the lice population) and CL_90_ (lethal concentration to kill 90% of the lice population), presented in Table 4, were determined from the regression line [mortality in probit = f(log concentration)] with corresponding 95% confidence intervals after 1 h and 2 h of exposure to the tested EO concentrations. The CL_50_ and CL_90_ values highlighted the insecticidal efficacy of the essential oils of *O. floribundum* and *E. citriodora* against *B. limbatus* lice.

The LC_50_ of oregano essential oil required to eliminate 50% of the lice population after 1 h of treatment is 0.410 µL/mL. After 2 h of exposure, it decreases to 0.112 µL/mL (Table 4). Similarly, a decrease in LC_50_ value with increasing exposure time was also observed for eucalyptus essential oil, with LC_50_ values of 0.319 µL/mL and 0.1 µL/mL found at 1 h and 2 h of treatment, respectively. The same applies to CL90 values for essential oils from *O. floribundum* and *E. citriodora*, which decrease from 3.54 µL/mL to 0.31 µL/mL and from 2.46 µL/mL to 0.24 µL/mL, respectively, after 1 h and 2 h of treatment. These results show a significant decrease in the LC_50_ and LC_90_ values of oregano and eucalyptus depending on the length of time the insects were exposed to the oils, suggesting that lice sensitivity increases in proportion to exposure time. A comparison of the lethal concentrations (LC_50_ and LC_90_) of oregano and eucalyptus essential oils shows that eucalyptus essential oil is slightly more toxic, suggesting that eucalyptus essential oil is more effective. However, statistical analysis comparing the lethal concentrations of oregano and eucalyptus essential oils found no significant differences (*p* > 5%), indicating comparable efficacy of the essential oils.

The calculation of the coefficients of determination from the regression curves gives an R^2^ with values between 0.87 and 0.99 (Table 4), indicating a very strong and proportional relationship between insecticidal activity and the concentration of the two essential oils.

The lethal time (LT_50_) and (LT_90_) values of both essential oils were negatively correlated with the concentrations tested (Table 5).

**Figure 4:**
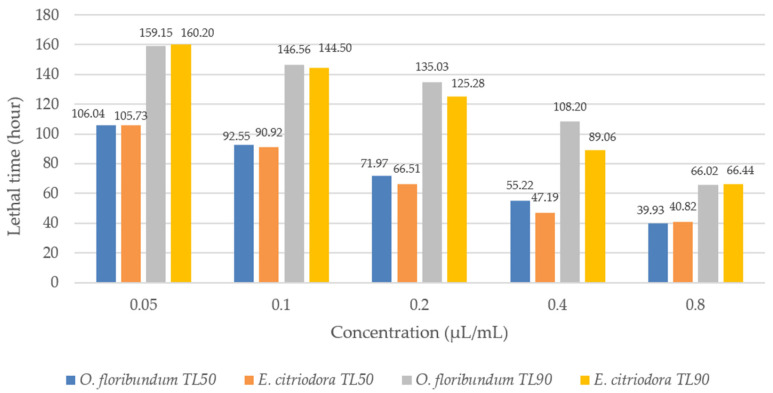
Lethal time causing the death of 50% (LT_50_) and 90% (LT_90_) of individuals of *Bovicola limbatus*.

Probit analysis showed that the sensitivity of the lice, *B. limbatus*, was proportional to the concentration of essential oils from both plants (Figure 4). Specifically, at a concentration of 0.8 μL/mL, the LT_50_ values for oregano and eucalyptus were 39.93 h and 40.82 h, respectively. Similarly, at the same concentration, LT_90_ values were 66.02 h for oregano and 66.44 h for eucalyptus.

## 3. Discussion

### 3.1. Yield and Chemical Composition

The yield of *O. floribundum* (2.86%) is very similar to that reported by Hadjadj and Hazzit (3%) [24] for a sample from the same location. However, Daoudi-Merbah et al. [17] observed a large variability in yield (2.5–5.6%) depending on the nature of the soil in this same location. *E. citriodora* EO yield (3.45) was very close to that recorded by Benchaa et al. (3.4%) [26].

### 3.2. Chromatographic Analysis by GC/FID and GC/SM

Chemical compositions of *O. floribundum* essential oils reported in the literature are characterized by either thymol [19,27], carvacrol [27], or p-cymene [17,24] as the most important compound. For *E. citridora*, our results are consistent with all studies reported in the literature that show citronellal as the most important compound [26,28,29], with differences in the amounts and order of other important compounds following citronellal. Differences could be principally attributed to the period of collection and maturity of the collected leaves, knowing that two studies, as well as the herein work, were made from the same trees.

### 3.3. Morphological Identification

The *B. limbatus* louse is mainly found on the skin of goats, feeding on skin debris and keratin, causing itching, bald patches or even inflammation in severe infestations [30]. However, even though a mild infestation generally has a limited and controlled effect on animal health, an unregulated parasite load can cause considerable discomfort, harm animal welfare and, in the long term, affect zootechnical performance [10,31].

The identification of both sexes is a biological indicator to be taken into consideration, as it reflects the active insect’s reproductive cycle. This observation indicates the need for regular monitoring of ectoparasite infestation in small ruminants, even in the absence of signs of disease. Research on *B. limbatus*, particularly in North Africa, is scarce [32]; however, several studies conducted in Europe confirm its presence in extensive goat farming, especially when preventive measures are not systematically applied [33]. This observation could be used for epidemiological research, including seasonal and environmental factors.

### 3.4. Contact Toxicity

The essential oil of *Eucalyptus citriodora*, long known in traditional medicine, is now described as an oil with strong potential for use as a biopesticide [34]. Its insecticidal efficacy against a wide range of harmful insects has been confirmed by numerous studies [34,35,36,37,38,39], including on insect populations resistant to chemical insecticides [40]. The results of this study have highlighted a particularly interesting contact toxicity of eucalyptus essential oil, probably due to these major compounds. Indeed, numerous studies [16,40] report the notable effectiveness of oil rich in citronellal, citronellol and isopulele, having a powerful repellent effect against the cosmopolitan stored product pest *Triboliuom castaneum* at a dose of 0.084 mL/L, an activity superior to that of the commercial product IR3535 [ethyl 3-(N-acetyl-N-butylamino)propionate] used at a dose of 0.686 mL/L [40]. This insecticidal action has also been demonstrated against *Lutzomyia longipalpis* at a concentration of 5 mg/mL [40]. Additionally, *E. citriodora* oil has shown larvicidal activity against *Culex quinquefasciatus* and acaricidal effects on larvae of *Amblyomma cajennense* and *Anocentor nitens* [41,42]. The synergy between its main components citronellal, citronellol, and isopuleol may explain its efficacy [40]. These constituents generally act synergistically, contributing to the overall pesticidal activity [34,43]. This effect could be due to the fact that, when the insect’s detoxification system targets the major component of the mixture, the secondary component poisons the insect and proves to be more toxic than when tested in isolation. Finally, beyond the immediate lethal effect, the oil’s constituents also disrupt various biochemical and physiological processes when ingested, inhaled or absorbed through the insect’s integument, leading to a gradual weakening of the organism, with notable effects on insect development and reproduction [14].

The results of toxicity tests on the essential oil of *O. floribundum* studied revealed significant insecticidal activity. This insecticidal activity is probably due to its chemical composition and mainly its major components. This essential oil is rich in monoterpenoids, known for their significant insecticidal activity against various insect species [44]. Indeed, chemical compounds known to have a broad spectrum of insecticidal activity include phenols (such as carvacrol), alcohols (such as linalool), aldehydes and ketones (such as citronellal) and monoterpene hydrocarbons (such as p-cymene) [45,46].

Yang et al., [47] describe the insecticidal activity of the essential oil of *Origanum majorana* L., which is more potent than two commonly used pediculicides (d-phenothrin or pyrethrum) against female *Pediculus humanus capitis* [47,48]. The work of Alahyane et al. [44,49], shows that the essential oils of *Thymus willdenowii* and *Thymus munbyanus*, rich in carvacrol, thymol and p-cymene, have acaricidal effects on *Varroa destructor*. In another study, treating bees heavily infested with *Varroa destructor* with *Thymus algeriensis* essential oil rich in carvacrol significantly reduced infestation rates to low levels in field trials [50]. This insecticidal activity is probably due to the synergistic action between carvacrol and other components of the essential oil, such as p-cymene [51,52]. Furthermore, the work of Tak and Isman [53] has shown that the cuticular penetration of essential oils and monoterpenes is enhanced when they are combined, thus the combination of thymol and p-cymene significantly increases their passage through the cuticle of insects, compared to the action of each individually [49].

Essential oils exert their insecticidal activity by targeting several molecular pathways in the insect nervous system. Certain molecules inhibit acetylcholinesterase, a key enzyme in nerve transmission, causing acetylcholine accumulation and paralysis. Other components act as allosteric modulators of GABA receptors, disrupting neuronal inhibition and inducing neurotoxic effects. In addition, many constituents also interact with insect-specific octopaminergic receptors, altering neurohormonal functions, motor skills and behaviour. This plurality of targets explains the effectiveness and broad spectrum of action of essential oils against various pests [54,55].

Pediculosis in ruminants often reflects a more serious underlying disease or problem, as the skins of sick or malnourished animals are damaged [30,32]. Mild infestations are generally well tolerated and go unnoticed, but losses can be considerable in severe infestations, which can affect the entire body surface and cause significant stress and itching [32]. Lice are the main ectoparasites responsible for skin disease in small ruminants and can cause serious economic losses to farmers, particularly due to the rejection of hides by the tanning industry [11,56]. All species cause pruritus, irritation and behavioural changes (licking, rubbing, agitation), which have a negative impact on growth and skin quality [11]. The control of ectoparasites using chemical insecticides in veterinary medicine is being called into question due to the development of resistance in insects [57]. Several essential oils have demonstrated in vitro and in vivo efficacy comparable to that of chemical insecticides against *Pediculus humanus* (human lice), serving as an experimental model for animal lice. Oils derived from eucalyptus, mint, rosemary, oregano, thyme, and ginger have rapid insecticidal properties, achieving mortality rates of 80 to 100% depending on concentration and formulation [48,58]. In poultry, a recent study has shown that combined formulations based on lemongrass and ginger effectively control lice and mites (100% mortality in vitro within 24 h and a 22.7% reduction in incidence within 14 days, comparable to trichlorfon used routinely) [59]. These essential oils show interesting potential for the development of innovative and safe products to combat infestations of ectoparasites, particularly lice.

## 4. Materials and Methods

### 4.1. Plant Material

Aerial parts of *O. floribundum* were collected in June 2023 in the leafing period from the Blida region (47 Km South West Algiers) at the National Park of Chrea (Coordinates: 36° 252 and 323 N, 2° 522 and 363 E, altitude: 1500 m), while the leaves of *E. citriodora* were collected at the same period from the botanical garden of the Higher National Agronomic School of Algiers (ENSA). The plants were compared to voucher already deposited in the herbarium of the botanical department and were authenticated by Pr. Hassen Abdelkrim (Plant taxonomist) from the Department of Botany of High National Agronomic School.

### 4.2. Essential Oil Extraction

The essential oil extraction was performed by hydrodistillation with a Clevenger-type apparatus. Shade-dried leaves (60 g of *O. floribundum* and 100 g of *E. citriodora*) were placed in 2 L flasks containing 1.5 L of distilled water. The extraction process lasted for 2 h. At the end of hydrodistillation, the essential oil was dried using anhydrous sodium sulphate and then stored at 4 °C, protected from light, for subsequent analyses.

### 4.3. Chromatographic Analysis by GC/FID and GC-SM

The essential oils were analyzed using GC and GC-MS. A GC-FID/7890 system was employed for gas chromatography (GC) analysis, equipped with a fused-silica capillary column and an apolar stationary phase DB 5MS (40 m × 0.18 mm × 0.18 μm film thickness). The temperature program for the column started at 50 °C for 5 min, then increased at a rate of 5 °C/min until reaching 300 °C, where it was maintained for 15 min. Samples (2 μL of 4% oil in hexane) were injected using a split injection with a split ratio of 1:100. The injection temperature was set at 280 °C, and the carrier gas (He) was delivered at a flow rate of 1.3 mL/min. Flame ionization detection was carried out at 320 °C. Quantitative data were directly obtained from the GC peak areas measured with the GC-FID, without applying any correction factors.

Gas chromatography coupled with electron ionization mass spectrometry (GC-MS) analyses were carried out using an automated system consisting of a 7890 gas chromatograph connected to a 5975C mass spectrometer. The setup employed an apolar DB 5MS column (40 m in length, 0.18 mm internal diameter, and 0.18 μm film thickness). The conditions for the GC-MS analysis included helium as the carrier gas at a flow rate of 1.3 mL/min, with a split ratio of 1:25. A sample volume of 0.2 μL (diluted 1/10 in hexane, *v*/*v*) was injected at an injector temperature of 280 °C. The oven temperature program used matched the one described previously for the gas chromatographic separation. Electron ionization was carried out at 70 eV, scanning masses in the range of 33 to 550 atomic mass units.

### 4.4. Identification of Components

Essential oil constituents were tentatively identified by comparison of their GC retention indices (RI), determined with reference to a homologous series of C8-C17 n-alkanes injected under the same conditions as the oils and RI from the literature [25]. Confirmation of such identification was done by comparing their mass spectral fragmentation patterns with those stored in the MS database (Library NIST 225,000 records) and with mass spectra literature data [25].

### 4.5. Lice Collection and Identification

The lice were collected from a herd of goats at the educational farm of the Higher National Veterinary School (ENSV), located in El Alia, Algiers, using a lice comb and stored in containers filled with 70% ethanol for preservation. The latter were taken to the zoology laboratory for identification. The clarification protocol followed these steps: immersion of the specimens in a potassium hydroxide solution (KOH 10%) for 24 h, followed by rinsing with distilled water for an additional 24 h. A series of ethanol baths at different concentrations (70%, 96% and 100%) was carried out. Finally, the lice were mounted between slide and coverslip with a drop of Canada balsam and placed in an oven at 60 °C for one week for fixation [60]. The samples were examined under an optical microscope (Leica DM 500, Leica Microsystems GmbH, Wetzlar, Germany). Species identification was carried out using dichotomous keys, in particular those proposed by Benítez-Rodríguez et al. [61], as well as resources from the specialist website Phthiraptera Info (https://phthiraptera.myspecies.info/category/chewing-lice/trichodectidae, accessed on 1 August 2025). The work was carried out with the assistance of Professor Marniche Faiza at the zoology laboratory (ENSV).

### 4.6. Contact Toxicity

The contact toxicity of the essential oils of *O. floribundum* and *E. citriodora* was evaluated using the filter paper method. The essential oils tested were prepared in acetone at the following concentrations: C_1_: 0.05 µL/mL; C_2_: 0.1 µL/mL; C_3_: 0.2 µL/mL; C_4_: 0.4 µL/mL; C_5_: 0.8 µL/mL, corresponding to the doses: D_1_: 0.0008 µL/cm^2^; D_2_: 0.0016 µL/cm^2^; D_3_: 0.0031 µL/cm^2^; D_4_: 0.0063 µL/cm^2^; D_5_: 0.0123 µL/cm^2^, respectively. Each prepared essential oil solution was evenly applied to a 9 cm diameter Whatman No. 1 filter paper, placed inside a Petri dish of the same diameter. The Petri dish was left open for 15 min to allow the evaporation of the dilution solvent. For the control group, the filter paper was treated with acetone only. A batch of twenty adult lice, collected from a goat herd, was introduced into each Petri dish. The dishes were then sealed with parafilm. Five replicates were performed for each concentration, including the control. Mortality was recorded at 15 min, 30 min, 1 h, 2 h, and 4 h post-exposure. The observed mortalities in treated Petri dishes (Mo) were corrected using Abbott’s formula [62], which accounts for natural mortality observed in the control dishes (Mt), according to the following equation:

(1) $$M_{c}=\frac{{(M}_{o}-M_{t})}{100-M_{t}}\times 100$$

M_c_: Corrected mortality; M_t_: Mortality in control group; M_o_: Mortality in treated group

### 4.7. Statistical Analyses

Statistical analyses were conducted using XLSTAT version 2019.1.2 (Addinsoft, Paris, France). Differences among treatments were assessed by analysis of variance (ANOVA), and where appropriate, pairwise comparisons of mean values for each concentration were carried out utilizing Tukey’s Honest Significant Difference (HSD) procedure. Results were considered statistically significant when the *p*-value was less than 0.05.

To determine the concentrations responsible for 50% and 90% mortality in the insect population, a probit analysis was used. This method involved converting the corrected mortality rates into probit values and performing a linear regression with the logarithmic concentrations tested. The same analytical approach was applied to estimate the time required to reach these lethal thresholds.

According to Finney [63], lethal dose and time values are derived from probit regression lines as a function of log_10_ dose and time. Similarly, LT_50_ and LT_90_ represent the time at which 50% and 90% of individuals have died.

## 5. Conclusions

In conclusion, the use of essential oils in the control of veterinary ectoparasites is a promising field with strong potential for the future. Indeed, essential oils from *O. floribundum* and *E. citriodora* have demonstrated significant contact toxicity against adult *B. limbatus* lice. However, research into their use as control agents is still at a preliminary stage. Large-scale field trials, toxicological studies on mammals, the development of appropriate excipients for direct application to the host, and in-depth investigation of the residual activity and shelf life of essential oils are essential before their full potential can be exploited.

## Figures and Tables

**Table 1:** Composition (%) of the essential oils of *Origanum floribundum* and *Eucalyptus citriodora*.

N°	Components	Retention Indices		Essential Oil	
		ERI	LRI	*Origanum floribundum*	*Eucalyptus citriodora*
1	α-Thujene	924	924	1.47	-
2	α-Pinene	934	932	0.63	0.13
3	Camphene	945	946	0.07	-
4	Sabinene	969	969	0.07	0.03
5	β-Pinene	974	974	0.15	0.72
6	1-Octen-3-ol	975	974	0.59	-
7	β-Myrcene	987	988	2.11	0.05
8	α-Phellandrene	1002	1002	0.20	-
9	δ-3-Carene	1008	1008	0.09	-
10	α-Terpinene	1016	1014	2.46	-
11	*p*-Cymene	1021	1020	**10.78**	0.03
12	Limonene	1024	1024	0.40	0.10
13	β-Phellandrene	1025	1025	0.30	0.01
14	Eucalyptol	1026	1026	0.02	0.41
15	*cis-*β-Ocimene	1030	1032	0.10	-
16	*trans*-β-Ocimene	1045	1044	0.04	-
17	γ-Terpinene	1054	1054	**18.91**	0.07
18	Melonal	1056	1056	-	0.48
19	*cis*-Sabinene hydrate	1063	1065	0.25	-
20	Terpinolene	1085	1086	0.04	-
21	Linalool	1095	1095	0.97	-
22	*trans*-Sabinene hydrate	1098	1098	0.15	-
23	*cis*-Rose oxide	1104	1106	-	1.19
24	*trans*-Rose oxide	1122	1122	-	0.09
25	Neo-isopulegol	1142	1144	-	0.54
26	Isopulegol	1144	1145	-	**18.59**
27	Citronellal	1148	1148	-	**58.01**
28	Borneol	1165	1165	0.06	-
29	4-Terpineol	1173	1174	0.20	0.08
30	α-Terpineol	1184	1186	0.26	0.04
31	*cis*-Dihydrocarvone	1192	1191	0.04	-
32	*trans*-Dihydrocarvone	1200	1200	0.02	-
33	Citronellol	1223	1223	-	**10.94**
34	Carvacrol methyl ether	1241	1241	1.61	-
35	Citronellyl formate	1270	1271	-	0.02
36	Thymol	1289	1289	2.16	-
37	Carvacrol	1301	1298	**54.63**	-
38	Citronellic acid	1312	1312	-	1.10
39	Citronellyl acetate	1352	1350	-	1.75
40	Phenylethyl isobutanoate	1351	1351	-	0.04
41	*cis*-Jasmone	1392	1392	-	0.14
42	β-Caryophyllene	1420	1417	0.59	0.16
43	α-Humulene	1451	1452	0.03	-
44	Germacrene D	1484	1484	0.01	-
45	Bicyclogermacrene	1500	1500	0.02	-
46	β-Bisabolene	1502	1505	0.06	-
47	δ-Cadinene	1521	1522	0.01	-
48	β-Sesquiphellandrene	1522	1522	0.14	-
49	Spathulenol	1577	1577	0.02	0.29
50	Caryophyllene oxide	1581	1582	0.06	0.69
51	γ-Eudesmol	1630	1630	-	0.02
	Total identification (%)			99.72	95.72
	Monoterpene hydrocarbons			37.82	1.14
	Oxygenated monoterpenes			60.37	93.38
	Sesquiterpene hydrocarbons			0.86	0.16
	Oxygenated sesquiterpenes			0.08	1.00
	Others			0.59	0.04
	*Oil yield % (v/w)*			2.86	3.45

Components are quantified on the DB5 MS capillary column and listed in order of their elution from the same column. ERI: Experimental retention indices relative to *n*-alkanes C8-C17 on DB5 MS column. LRI: Literature retention indices relative to DB5 column [25]; (-): Not detected. Values in bold mean main components.

**Table 2:** Mortality rate of the insecticidal activity of *Origanum floribundum* and *Eucalyptus citriodora* EO against *Bovicola limbatus*.

Tested EO	C * (μL/mL)	Mean Mortality Rates (%, ±SD) at Different Treatment Times				
		15 min	30 min	1 h	2 h	4 h
*Origanum floribundum*	C_1_: 0.05	0% ± 0%	0% ± 0%	10% ± 3%	26% ± 2%	100% ± 0%
	C_2_: 0.1	0% ± 0%	0% ± 0%	22% ± 2%	44% ± 5%	100% ± 0%
	C_3_: 0.2	0% ± 0%	8% ± 2%	32% ± 4%	55% ± 6%	100% ± 0%
	C_4_: 0.4	0% ± 0%	18% ± 2%	49% ± 2%	87% ± 4%	100% ± 0%
	C_5_: 0.8	0% ± 0%	33% ± 2%	66% ± 2%	100% ± 0%	100% ± 0%
	Control	0% ± 0%	0% ± 0%	0% ± 0%	0% ± 0%	0% ± 0%
*Eucalyptus citriodora*	0.05	0% ± 0%	0% ± 0%	12% ± 4%	23% ± 2%	100% ± 0%
	0.1	0% ± 0%	0% ± 0%	23% ± 0%	48% ± 2%	100% ± 0%
	0.2	0% ± 0%	9% ± 2%	40% ± 0%	68% ± 2%	100% ± 0%
	0.4	0% ± 0%	20% ± 0%	56% ± 0%	99% ± 2%	100% ± 0%
	0.8	0% ± 0%	28% ± 2%	71% ± 0%	100% ± 0%	100% ± 0%
	Control	0% ± 0%	0% ± 0%	0% ± 0%	0% ± 0%	0% ± 0%

C *: Concentration.

**Table 3:** Analysis of variance of factors influencing the mortality rate of *Bovicola limbatus* treated with EO.

Source	ddl	Somme Squared	Mean Squared	F Value	Pr > F
Extract	1.000	0.004	0.004	0.266	0.609
Time	4.000	6.592	1.648	99.315	<0.0001
Concentration	4.000	0.723	0.181	10.889	<0.0001

**Table 4:** Toxicity parameters of the essential oils of *Origanum floribundum* and *Eucalyptus citriodora* on *Bovicola limbatus*.

Essential Oil	Exposure Time (h)	Regression Equation	R^2^	LC_50_(95%CI) (μL/mL)	LC_90_(95%CI) (μL/mL)	χ^2^	ddl	*p*-Value
*Origanum floribundum*	1	y = 5.529 + 1.369x	0.995	0.410 [0.284–0.596]	3.54 [2.828–4.438]	0.0018	3	>0.05
*Origanum floribundum*	2	y = 7.741 + 2.903x	0.874	0.112 [0.095–0.136]	0.31 [0.282–0.349]	0.1834	3	>0.05
*Eucalyptus citriodora*	1	y = 5.716 + 1.445x	0.998	0.319 [0.225–0.452]	2.46 [1.81–3.30]	0.0009	3	>0.05
*Eucalyptus citriodora*	2	y = 8.351 + 3.335x	0.956	0.1 [0.081–0.119]	0.24 [0.218–0.262]	0.0808	3	>0.05

R^2^: coefficient of determination; IC_95%_: Confidence intervals; LC_50_ and LC_90_: Lethal concentrations for 50% and 90%.

**Table 5:** LT_50_ and LT_90_ of *Bovicola limbatus* adults exposed to *Origanum floribundum* and *Eucalyptus citriodora* EOs.

Concentration EO of *Origanum floribundum* (μL/mL)	LT_50_ *	r	LT_90_ *	r
0.05	106.038	−0.928	159.150	−0.996
0.1	92.549		146.561	
0.2	71.970		135.029	
0.4	55.221		108.202	
0.8	39.934		66.017	
Concentration EO of *Eucalyptus citriodora* (μL/mL)	LT_50_	r	LT_90_	r
0.05	105.729	−0.873	160.204	−0.955
0.1	90.920		144.500	
0.2	66.506		125.284	
0.4	47.192		89.060	
0.8	40.815		66.443	

* LT_50_ and LT_90_: Lethal time for 50% and 90%.

## Data Availability

The original contributions presented in this study are included in the article/Supplementary Materials. Further inquiries can be directed to the corresponding author.

## Supplementary Materials

The following supporting information can be downloaded at: https://www.mdpi.com/article/10.3390/molecules30194001/s1, Chromatograms, GC-MS analysis and mass spectra of the main components of the essential oils.
