# Supplementary material for: Chemical Composition and Insecticidal Activity of Essential Oils from Origanum floribundum and Eucalyptus citriodora Against the Louse Bovicola limbatus
Source: Molecules. 2025 Oct 6;30(19):4001. doi: 10.3390/molecules30194001 (PMC12525578; doi:10.3390/molecules30194001)

```
Print of window 38: Current Chromatogram(s)
Data File   : S:\G220721-HUILE\G-71901.D
Sample Name : Origanum floribundum -
======================================================================
Acq. Operator   : VB
                                                   Location : Vial 17
Injection Date  : 07/22/22 8:24:00 AM
Acq. Method     : LIQ-HP5-40-HUILE.M
Analysis Method : R:\GC-G\7890\MEGA\HE\ORIGAN-COMPACT-CAS.M
Last changed    : 07/22/22 10:15:11 AM
                  (modified after loading)
Additional Info : Peak(s) manually integrated
```

Current Chromatogram(s)

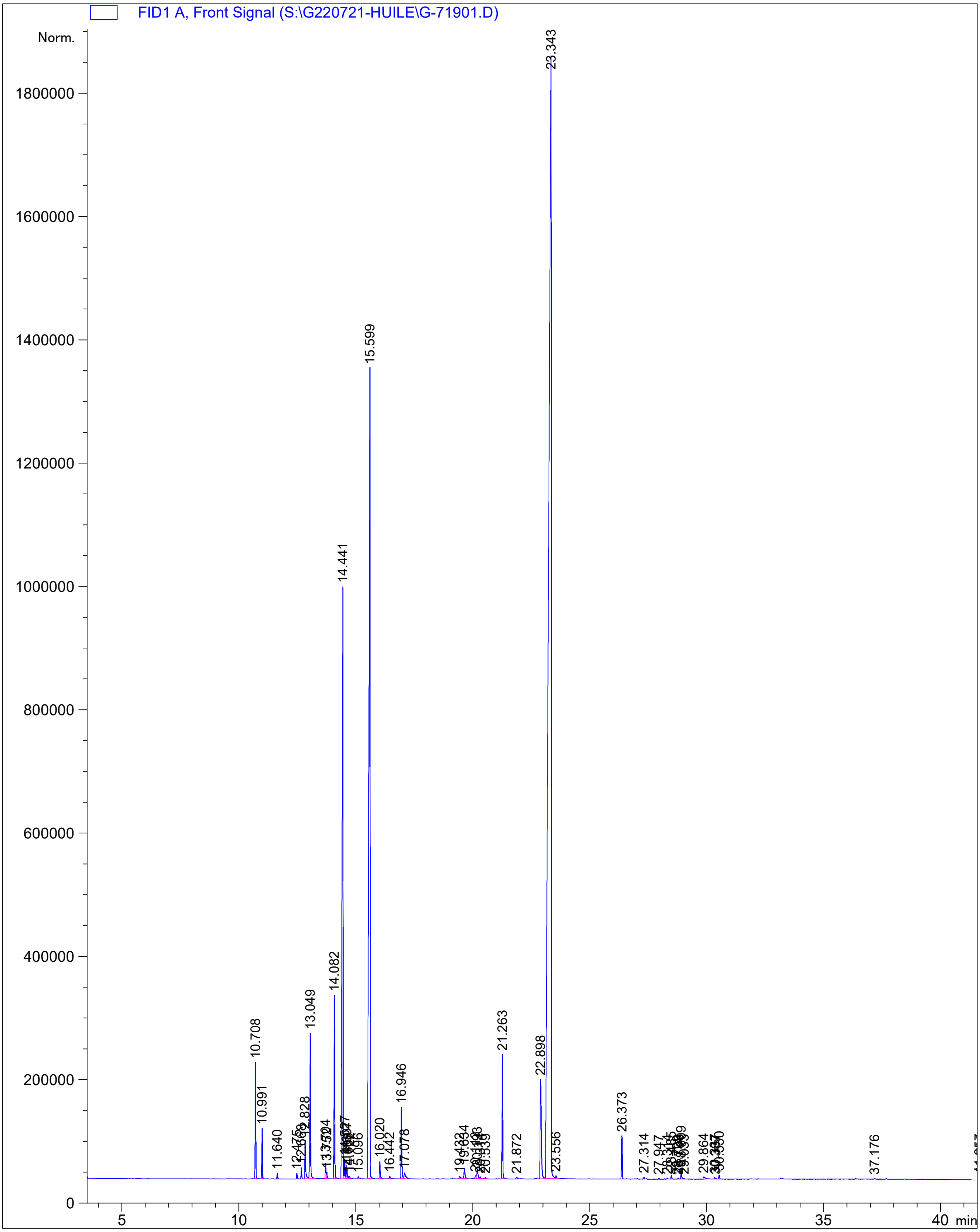

Supplement: Supplementary file 1 [file molecules-30-04001-s001.zip › 71901 Origanum floribundum Chromato ENSV 2.pdf]
