# Supplementary material for: Chemical Composition and Insecticidal Activity of Essential Oils from Origanum floribundum and Eucalyptus citriodora Against the Louse Bovicola limbatus
Source: Molecules. 2025 Oct 6;30(19):4001. doi: 10.3390/molecules30194001 (PMC12525578; doi:10.3390/molecules30194001)
Supplement: Supplementary file 1 [file molecules-30-04001-s001.zip › 71901a Origan ENSV 2.pdf]

ENSV El-Harrach
Mme Nassima CHORFI
13 Lot les castors groupe 1
16000 ALGER
Algérie

Référence de la commande : DA du 19/07/2022
Date de réception de l'échantillon : 21/07/2022
Version document : ENR-047-V2 du 01/08/2021

BULLETIN D'ANALYSE N°: 71901

Origanum floribundum

Désignation de l'échantillon : Origanum floribundum

Nom botanique : Origanum floribundum

Analyse chromatographique par GC/FID

Préparation échantillon : Dilution au 50ème dans l'hexane

Tr	N° CAS	Composés	% Fid
10.71	2867-05-2	Alpha-Thujène	1.474
10.99	80-56-8	Alpha-Pinène	0.628
11.64	79-92-5	Camphène	0.073
12.48	3387-41-5	Sabinène	0.072
12.67	127-91-3	Béta-Pinène	0.145
12.83	3391-86-4	1-Octèn-3-ol	0.587
13.05	123-35-3	Myrcène	2.112
13.70	99-83-2	Alpha-Phellandrène	0.204
13.75	13466-78-9	Delta-3-Carène	0.090
14.08	99-86-5	Alpha-Terpinène	2.463
14.44	99-87-6	Para-Cymène	10.777
14.53	138-86-3	Limonène	0.404
14.61	555-10-2	Béta-Phellandrène	0.298
14.67	470-82-6	Eucalyptol	0.023
14.72	3338-55-4	(Z)-Béta-Ocimène	0.095
15.10	3779-61-1	(E)-Béta-Ocimène	0.038
15.60	99-85-4	Gamma-Terpinène	18.913
16.02	17699-16-0	Cis-Hydrate de Sabinène	0.245
16.44	586-62-9	Terpinolène	0.036
16.95	78-70-6	Linalol	0.974
17.08	15826-82-1	Trans-Hydrate de Sabinène (OH vs IPP)	0.147
19.43	507-70-0	Bornéol	0.062
19.63	562-74-3	Terpinène-4-ol	0.203

Saint Beauzire le 28/07/2022 17:09
Dr. Gilles FIGUEREDO
Directeur du laboratoire

LEXVA Analytique

Référence de la commande : DA du 19/07/2022
Date de réception de l'échantillon : 21/07/2022
Version document : ENR-047-V2 du 01/08/2021

ENSV El-Harrach
Mme Nassima CHORFI
13 Lot les castors groupe 1
16000 ALGER
Algérie

BULLETIN D'ANALYSE N°: 71901

Origanum floribundum

Analyse chromatographique par GC/FID (suite)

Tr	N° CAS	Composés	% Fid
20.11	-	Alpha-Terpinéol*	0.062
20.19	98-55-5	Alpha-Terpinéol	0.201
20.31	7764-50-3	Cis-Dihydrocarvone	0.035
20.54	5948-04-9	Trans Dihydro carvone	0.023
21.26	6379-73-3	Carvacrol Méthyl Ether	1.608
22.90	89-83-8	Thymol	2.161
23.34	499-75-2	Carvacrol	54.630
26.37	87-44-5	Béta-Caryophyllène	0.591
27.31	6753-98-6	Alpha-Humulène	0.029
27.95	23986-74-5	Germacrène D	0.011
28.31	24703-35-3	Bicyclogermacrène	0.016
28.49	495-61-4	Béta Bisabolène	0.056
28.73	39029-41-9	Gamma-Cadinène	0.004
28.80	483-76-1	Delta-Cadinène	0.012
28.91	20307-83-9	Béta-Sesquiphellandrène	0.142
30.34	6750-60-3	Spathulénol	0.020
30.53	1139-30-6	Oxyde de Caryophyllène	0.063
		Total	99.728

* Isomère non identifié

Saint Beauzire le 28/07/2022 17:09
Dr. Gilles FIGUEREDO
Directeur du laboratoire

ENSV El-Harrach
Mme Nassima CHORFI
13 Lot les castors groupe 1
16000 ALGER
Algérie

Référence de la commande : DA du 19/07/2022
Date de réception de l'échantillon : 21/07/2022
Version document : ENR-047-V2 du 01/08/2021

BULLETIN D'ANALYSE N°: 71901

Origanum floribundum

Conditions opératoires/ Operating conditions

Version française	English version
**Chromatographe gazeux : CPG/FID 7890**	**Gaz chromatograph : CPG/FID 7890**
Colonne Apolaire : DB5 MS : 40 m 0,18 mm 0,18 µm	Colonn Apolar : DB5 MS : 40 m 0,18 mm 0,18 µm
Programmation de température : 50 °C pdt 5 min – 5 °C/min °C jusqu'à 300 °C	Temperature Programming 50 °C for 5min – 5 °C/min °C – until 300 °C
Gaz vecteur : He : 1.3 ml/min	Gas vector : He : 1.3 ml/min
Échantillon : 4% en solution dans l'acétone ou l'hexane	Sample : 4% of solution in acetone or hexane
Volume d'injection : 2 µl	Injection volume : 2 µl
Injecteur : 280 °C avec diviseur 1/100	Injector : 280 °C with split 1/100
Les % sont calculés à partir des surfaces de pics donnés par le GC/FID sans l'utilisation de facteur de correction	The % is calculated from the peaks area given by the GC/FID without the use of correction factor
**Chromatographe gazeux : CPG/MS 7890/5975C**	**Gaz chromatograph : CPG/MS 7890/5975C**
Colonne : Apolaire : DB5 MS : 40 m 0,18 mm 0,18 µm	Colonn : Apolar : DB5 MS : 40 m 0,18 mm 0,18 µm
Programmation de température : 50 °C pdt 5 min – 5 °C/min °C jusqu'à 300 °C	Temperature Programming 50 °C for 5min – 5 °C/min °C – until 300 °C
Gaz vecteur : He : 1.3 ml/min	Gas vector : He : 1.3 ml/min
Échantillon : 4% en solution dans l'acétone ou l'hexane	Sample : 4% of solution in acetone or hexane
Volume d'injection : 2 µL	Injection volume : 2 µL
Injecteur : 280 °C avec diviseur 1/100	Injector : 280 °C with split 1/100
Gamme de masse : 33 à 550	Mass Range : 33 at 550
Les composés de l'huile sont identifiés par une recherche combinée des temps de rétention (bibliothèque du laboratoire) et des spectres de masse (librairie NIST 225 000 spectres)	The components of the oil are identified by a combined search of retention times (lab library) and mass spectra (Library NIST 225000 records).

Saint Beauzire le 28/07/2022 17:09
Dr. Gilles FIGUEREDO
Directeur du laboratoire
