# Supplementary material for: Chemical Composition and Insecticidal Activity of Essential Oils from Origanum floribundum and Eucalyptus citriodora Against the Louse Bovicola limbatus
Source: Molecules. 2025 Oct 6;30(19):4001. doi: 10.3390/molecules30194001 (PMC12525578; doi:10.3390/molecules30194001)

Print of window 38: Current Chromatogram(s)
Data File : S:\G220721-HUILE\G-71902.D
Sample Name : Eucalyptus citriodora -

===

Acq. Operator : VB
Location : Vial 18
Injection Date : 07/22/22 9:26:00 AM
Acq. Method : LIQ-HP5-40-HUILE.M
Analysis Method : R:\GC-G\7890\MEGA\HE\EUCALYPTUS CITRON-CAS.M
Last changed : 07/22/22 2:27:34 PM
(modified after loading)
Additional Info : Peak(s) manually integrated

Current Chromatogram(s)

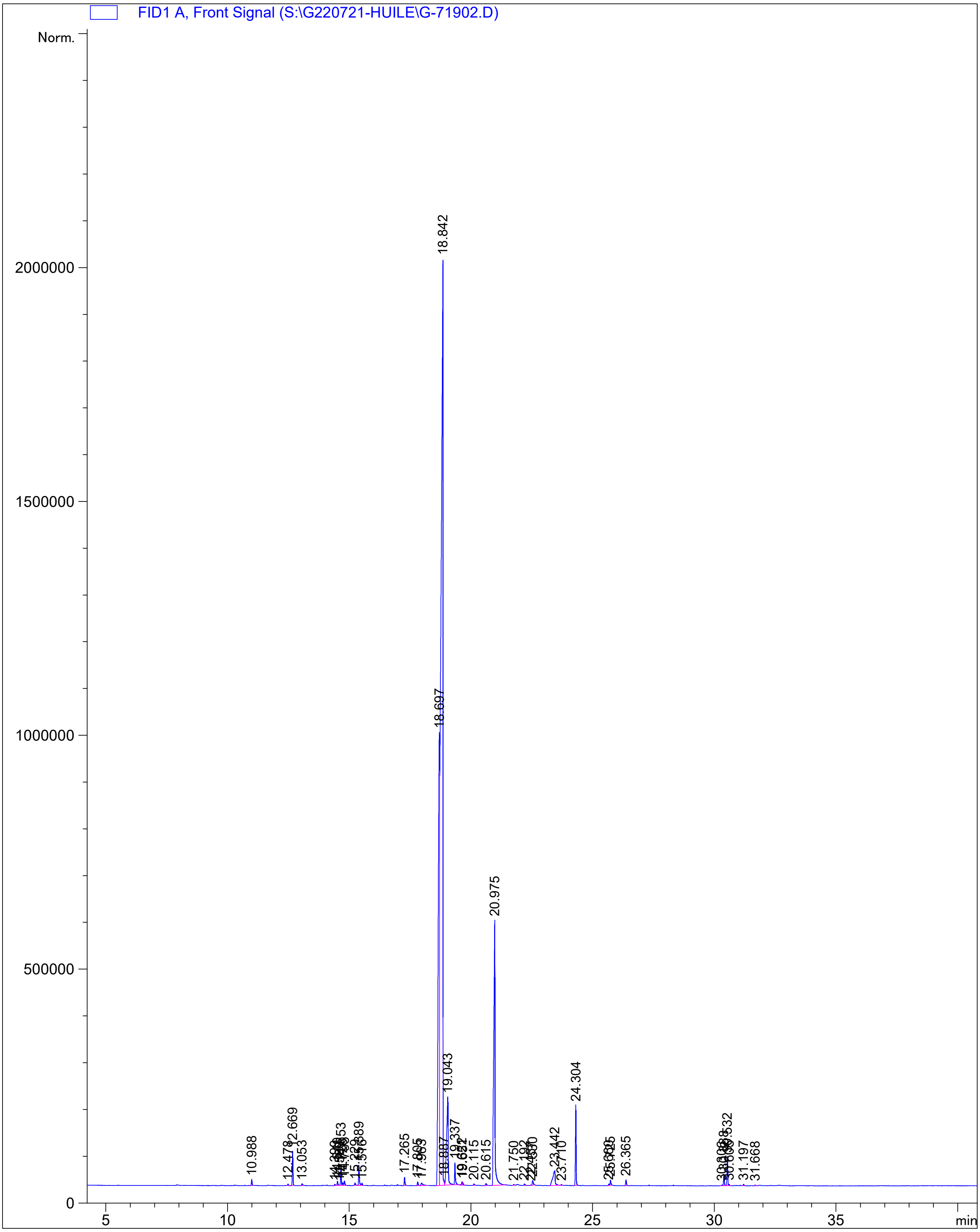

Supplement: Supplementary file 1 [file molecules-30-04001-s001.zip › 71902 Eucalyptus Chromato ENSV 2.pdf]
