# Supplementary material for: Chemical Composition and Insecticidal Activity of Essential Oils from Origanum floribundum and Eucalyptus citriodora Against the Louse Bovicola limbatus
Source: Molecules. 2025 Oct 6;30(19):4001. doi: 10.3390/molecules30194001 (PMC12525578; doi:10.3390/molecules30194001)
Supplement: Supplementary file 1 [file molecules-30-04001-s001.zip › 71902a Eucalyptus citronne ENSV 2.pdf]

Référence de la commande : DA du 19/07/2022
Date de réception de l'échantillon : 21/07/2022
Version document : ENR-047-V2 du 01/08/2021

ENSV El-Harrach
Mme Nassima CHORFI
13 Lot les castors groupe 1
16000 ALGER
Algérie

BULLETIN D'ANALYSE N°: 71902

Eucalyptus citriodora

Nom botanique : Eucalyptus citriodora

Analyse chromatographique par GC/FID

Préparation échantillon : Dilution au 50ème dans l'hexane

Tr	N° CAS	Composés	% Fid
10.99	80-56-8	Alpha-Pinène	0.126
12.48	3387-41-5	Sabinène	0.033
12.67	127-91-3	Béta-Pinène	0.722
13.05	123-35-3	Myrcène	0.049
14.40	99-87-6	Para-Cyméne	0.034
14.51	138-86-3	Limonène	0.099
14.60	555-10-2	Béta-Phellandrène	0.014
14.65	470-82-6	Eucalyptol	0.412
14.73	-	Monoterpène Oxygéné Masse molaire 154	0.068
14.79	-	Monoterpène Oxygéné Masse molaire 154	0.120
15.39	106-72-9	Mélonal	0.481
15.52	99-85-4	Gamma-Terpinène	0.065
17.27	4610-11-1	Cis-Oxyde de Rose	0.192
17.81	5258-11-7	Trans-Oxyde de Rose	0.091
17.96	4234-93-9	2,6-Diméthyl-5-Heptène-1-ol	0.150
18.70	89-79-2	Isopulégol	18.587
18.84	106-23-0	Citronellal	58.005
19.04	18674-65-2	Iso-Isopulégol	4.417
19.34	21290-09-5	Néo-Iso-Isopulégol	0.542
19.62	562-74-3	Terpinène-4-ol	0.083
20.12	98-55-5	Alpha-Terpinéol	0.037
20.97	106-22-9	Citronnellol	10.935
21.75	2270-60-2	Citronellate de Méthyle	0.023
23.44	502-47-6	Acide Citronellique	1.098
24.30	150-54-5	Acétate de Citronnellyle	1.754

Saint Beauzire le 28/07/2022 17:10
Dr. Gilles FIGUEREDO
Directeur du laboratoire

LEXVA *Analytique* - 7 Rue Henri Mondor - Biopôle Clermont-Limagne - 63360 Saint Beauzire
Téléphone : 09 67 31 60 63 - Fax : 04 73 97 60 63 - e-mail : contact@lexva-analytique.com
SARL au capital de 10 000 euros - R.C.S. CLERMONT FERRAND 495 337 529 -N° TVA FR 50 495 337 529

Référence de la commande : DA du 19/07/2022
Date de réception de l'échantillon : 21/07/2022
Version document : ENR-047-V2 du 01/08/2021

ENSV El-Harrach
Mme Nassima CHORFI
13 Lot les castors groupe 1
16000 ALGER
Algérie

BULLETIN D'ANALYSE N°: 71902

Eucalyptus citriodora

Analyse chromatographique par GC/FID (suite)

Tr	N° CAS	Composés	% Fid
25.66	7775-39-5	Isobutanoate de Phényl Ethyle	0.038
25.73	488-10-8	(Z)-Jasmone	0.137
26.37	87-44-5	Béta-Caryophyllène	0.155
30.31	-	Sesquiterpène Oxygéné Masse molaire 222	0.019
30.39	6750-60-3	Spathulénol	0.286
30.53	1139-30-6	Oxyde de Caryophyllène	0.686
31.67	1209-71-8	Gamma-Eudesmol	0.020
		Total	99.478

Saint Beauzire le 28/07/2022 17:10
Dr. Gilles FIGUEREDO
Directeur du laboratoire

ENSV El-Harrach
Mme Nassima CHORFI
13 Lot les castors groupe 1
16000 ALGER
Algérie

Référence de la commande : DA du 19/07/2022
Date de réception de l'échantillon : 21/07/2022
Version document : ENR-047-V2 du 01/08/2021

BULLETIN D'ANALYSE N°: 71902

Eucalyptus citriodora

Conditions opératoires/ Operating conditions

Version française	English version
**Chromatographe gazeux : CPG/FID 7890**	**Gaz chromatograph : CPG/FID 7890**
Colonne Apolaire : DB5 MS : 40 m 0,18 mm 0,18 µm	Colonn Apolar : DB5 MS : 40 m 0,18 mm 0,18 µm
Programmation de température : 50 °C pdt 5 min – 5 °C/min °C jusqu'à 300 °C	Temperature Programming 50 °C for 5min – 5 °C/min °C – until 300 °C
Gaz vecteur : He : 1.3 ml/min	Gas vector : He : 1.3 ml/min
Échantillon : 4% en solution dans l'acétone ou l'hexane	Sample : 4% of solution in acetone or hexane
Volume d'injection : 2 µl	Injection volume : 2 µl
Injecteur : 280 °C avec diviseur 1/100	Injector : 280 °C with split 1/100
Les % sont calculés à partir des surfaces de pics donnés par le GC/FID sans l'utilisation de facteur de correction	The % is calculated from the peaks area given by the GC/FID without the use of correction factor
**Chromatographe gazeux : CPG/MS 7890/5975C**	**Gaz chromatograph : CPG/MS 7890/5975C**
Colonne : Apolaire : DB5 MS : 40 m 0,18 mm 0,18 µm	Colonn : Apolar : DB5 MS : 40 m 0,18 mm 0,18 µm
Programmation de température : 50 °C pdt 5 min – 5 °C/min °C jusqu'à 300 °C	Temperature Programming 50 °C for 5min – 5 °C/min °C – until 300 °C
Gaz vecteur : He : 1.3 ml/min	Gas vector : He : 1.3 ml/min
Échantillon : 4% en solution dans l'acétone ou l'hexane	Sample : 4% of solution in acetone or hexane
Volume d'injection : 2 µL	Injection volume : 2 µL
Injecteur : 280 °C avec diviseur 1/100	Injector : 280 °C with split 1/100
Gamme de masse : 33 à 550	Mass Range : 33 at 550
Les composés de l'huile sont identifiés par une recherche combinée des temps de rétention (bibliothèque du laboratoire) et des spectres de masse (librairie NIST 225 000 spectres)	The components of the oil are identified by a combined search of retention times (lab library) and mass spectra (Library NIST 225000 records).

Saint Beauzire le 28/07/2022 17:10
Dr. Gilles FIGUEREDO
Directeur du laboratoire
