# Supplementary material for: Chemical Composition and Insecticidal Activity of Essential Oils from Origanum floribundum and Eucalyptus citriodora Against the Louse Bovicola limbatus
Source: Molecules. 2025 Oct 6;30(19):4001. doi: 10.3390/molecules30194001 (PMC12525578; doi:10.3390/molecules30194001)
Supplement: Supplementary file 1 [file molecules-30-04001-s001.zip › Mass spectra.pdf]

Mass spectra of the main components of *Eucalyptus citriodora* essential oil

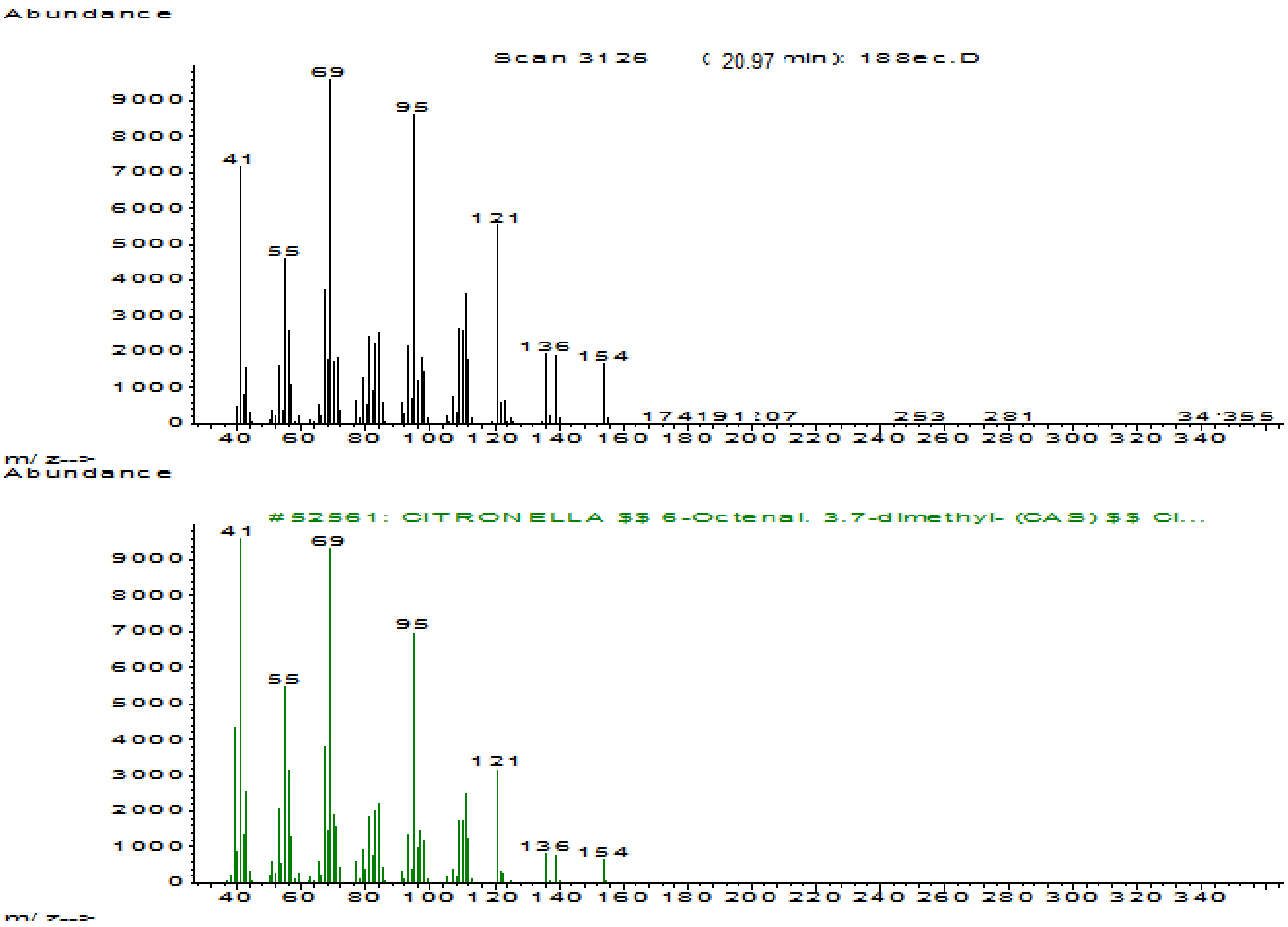

Citronellal

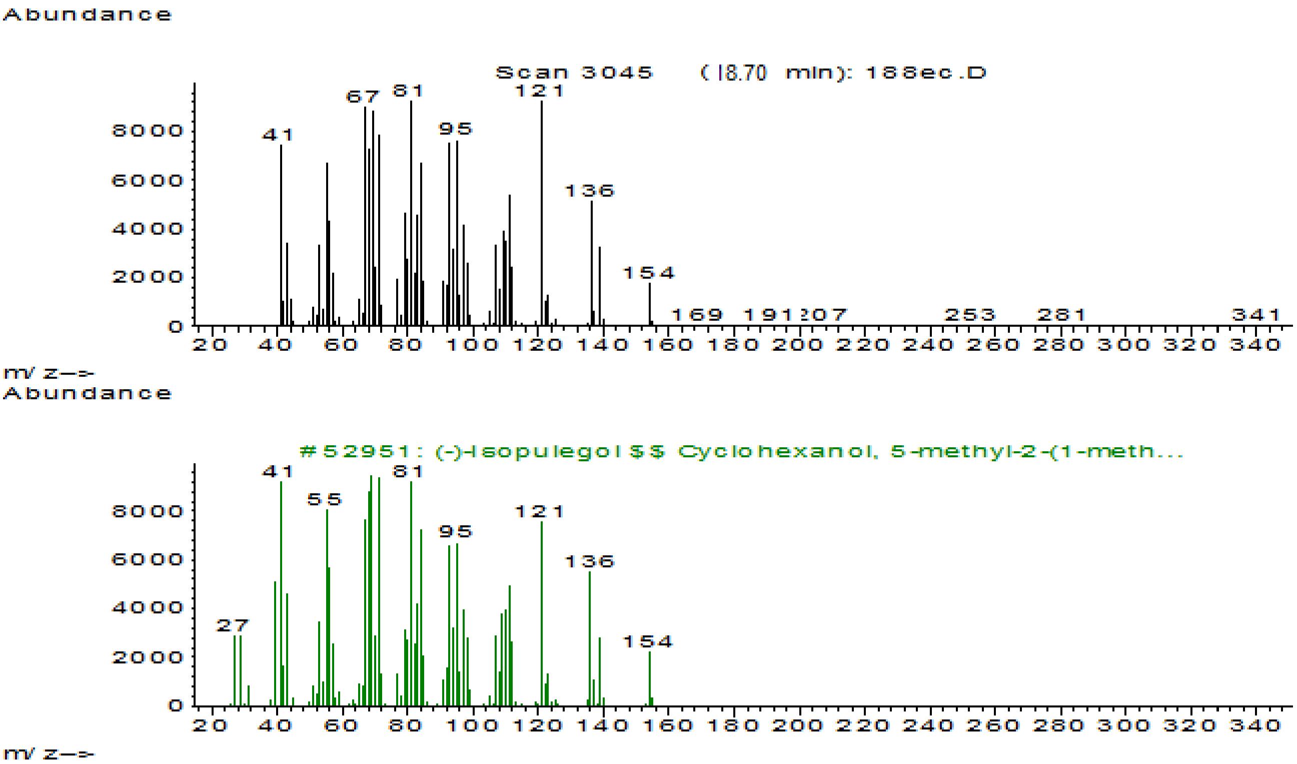

Isopulegol

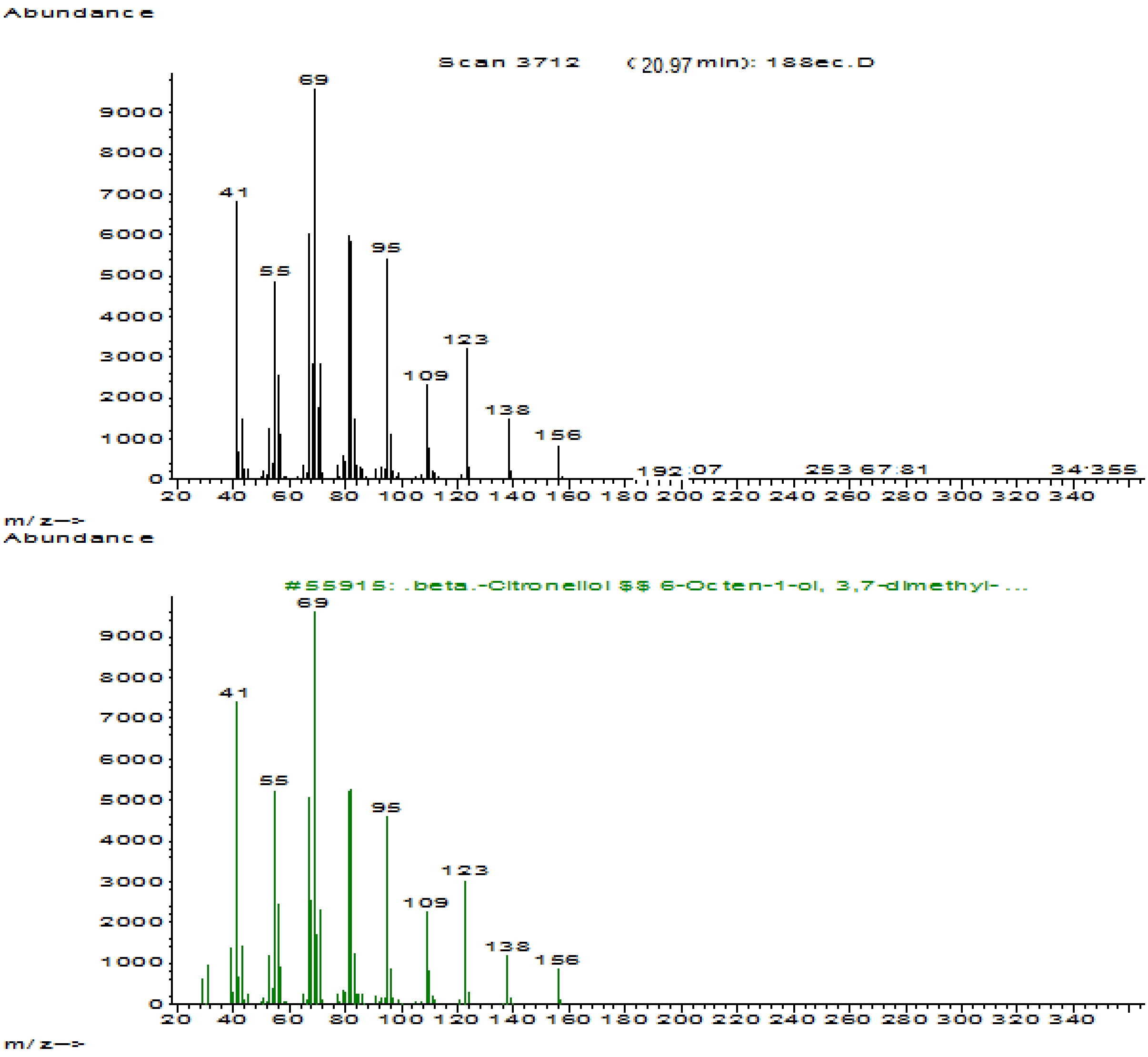

Citronellol

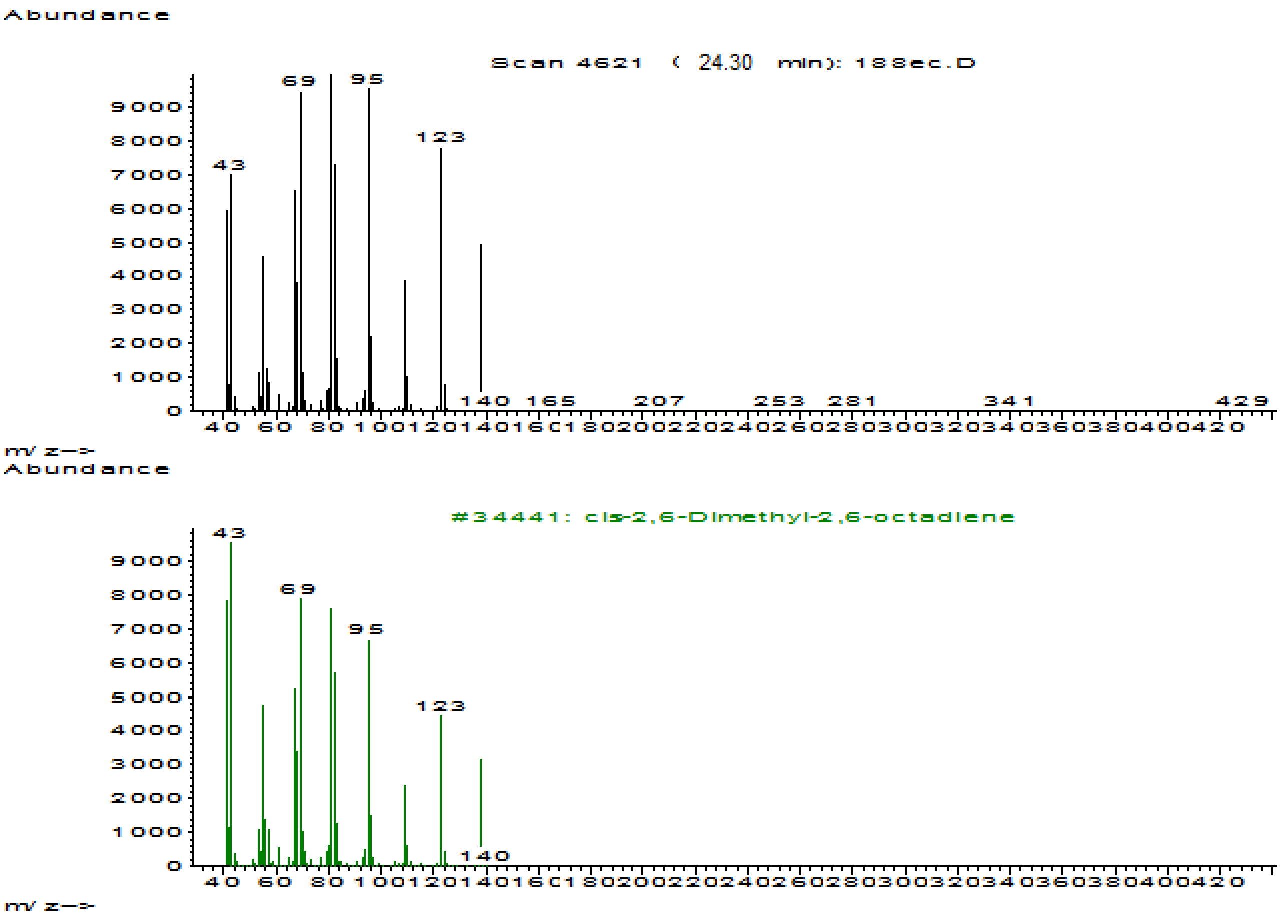

Citronellyl acetate

Mass spectra of the main components of *Origanum floribundum* essential oil

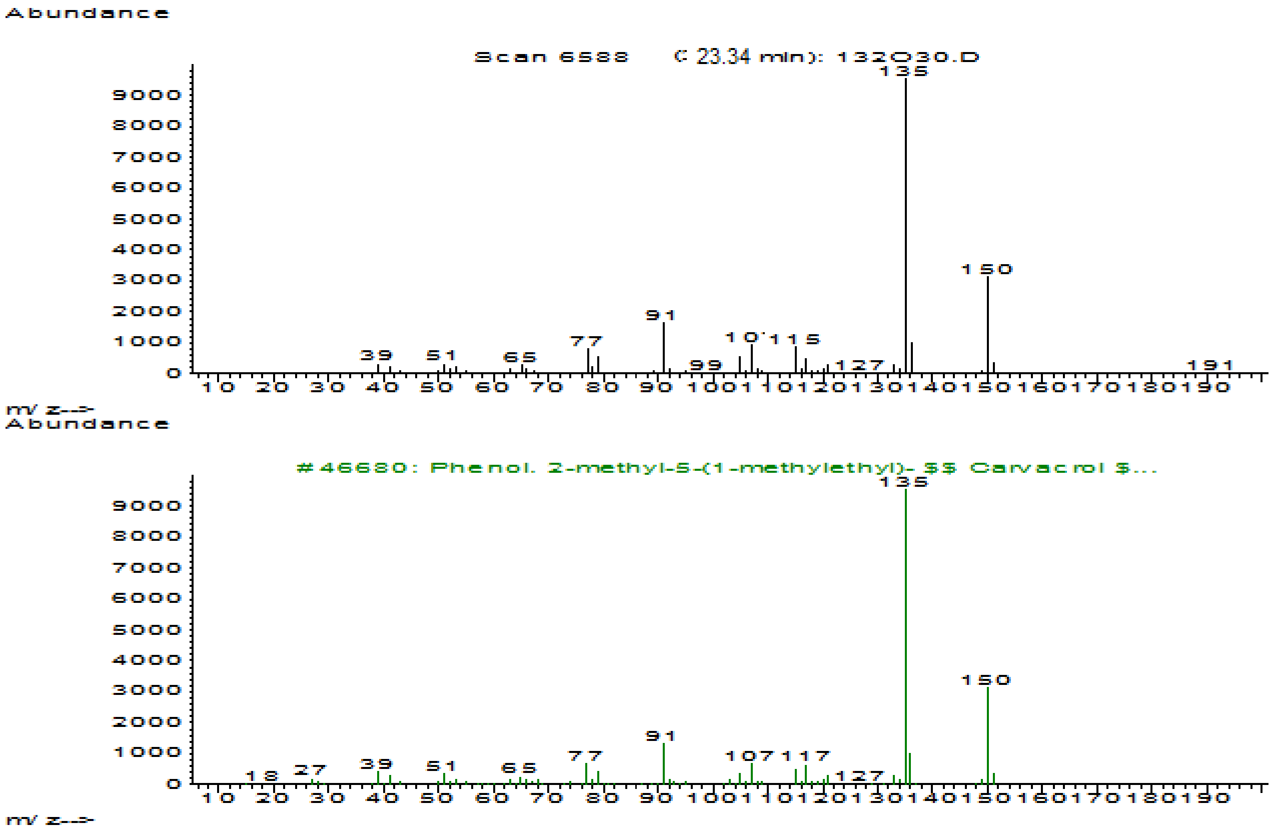

Carvacrol

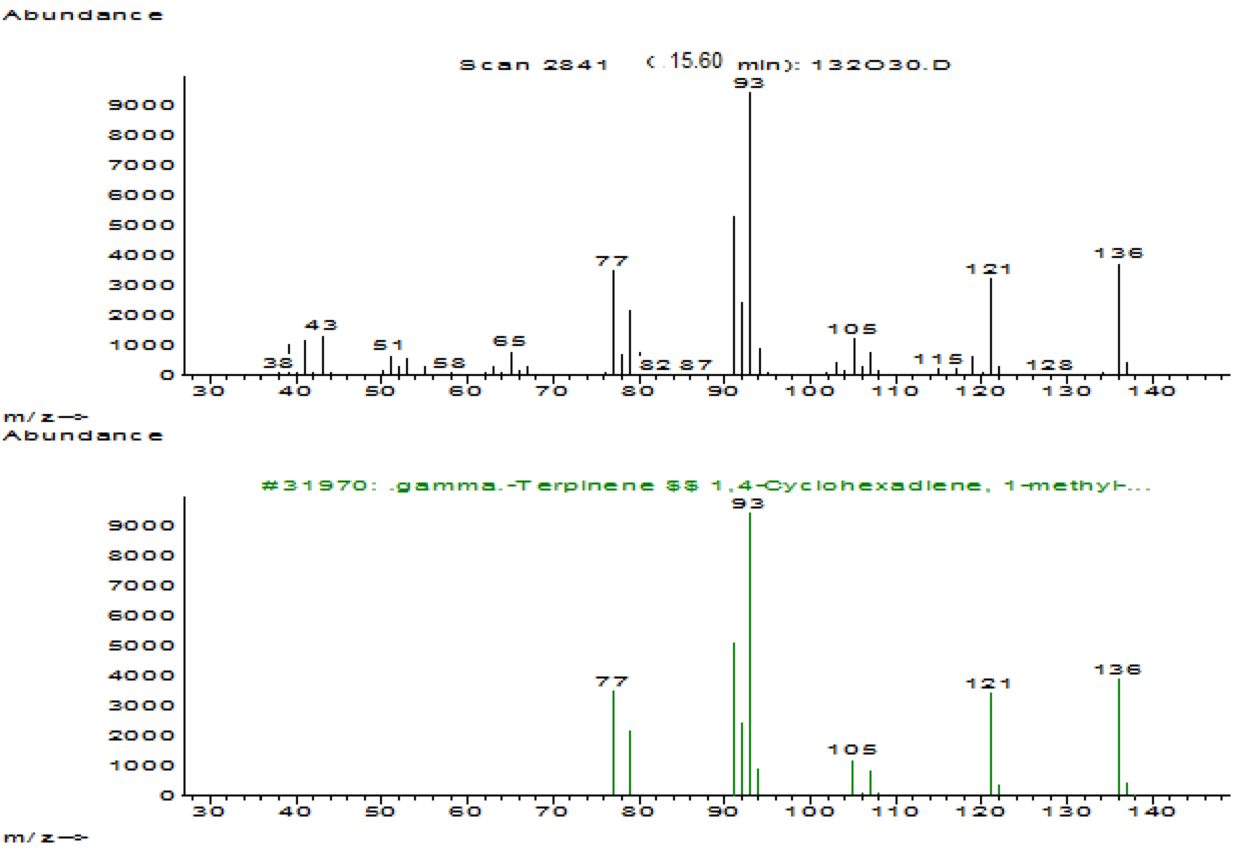

γ-Terpinene

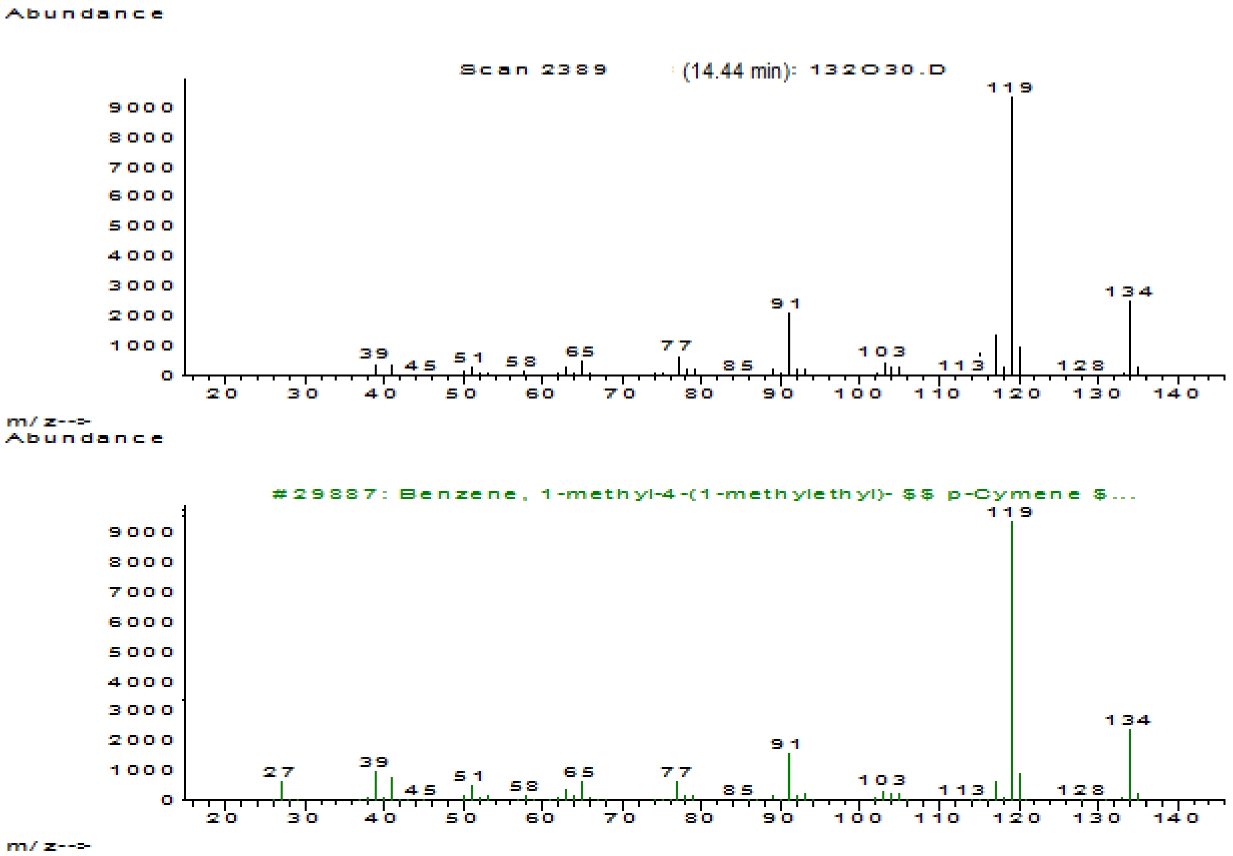

p-Cymene

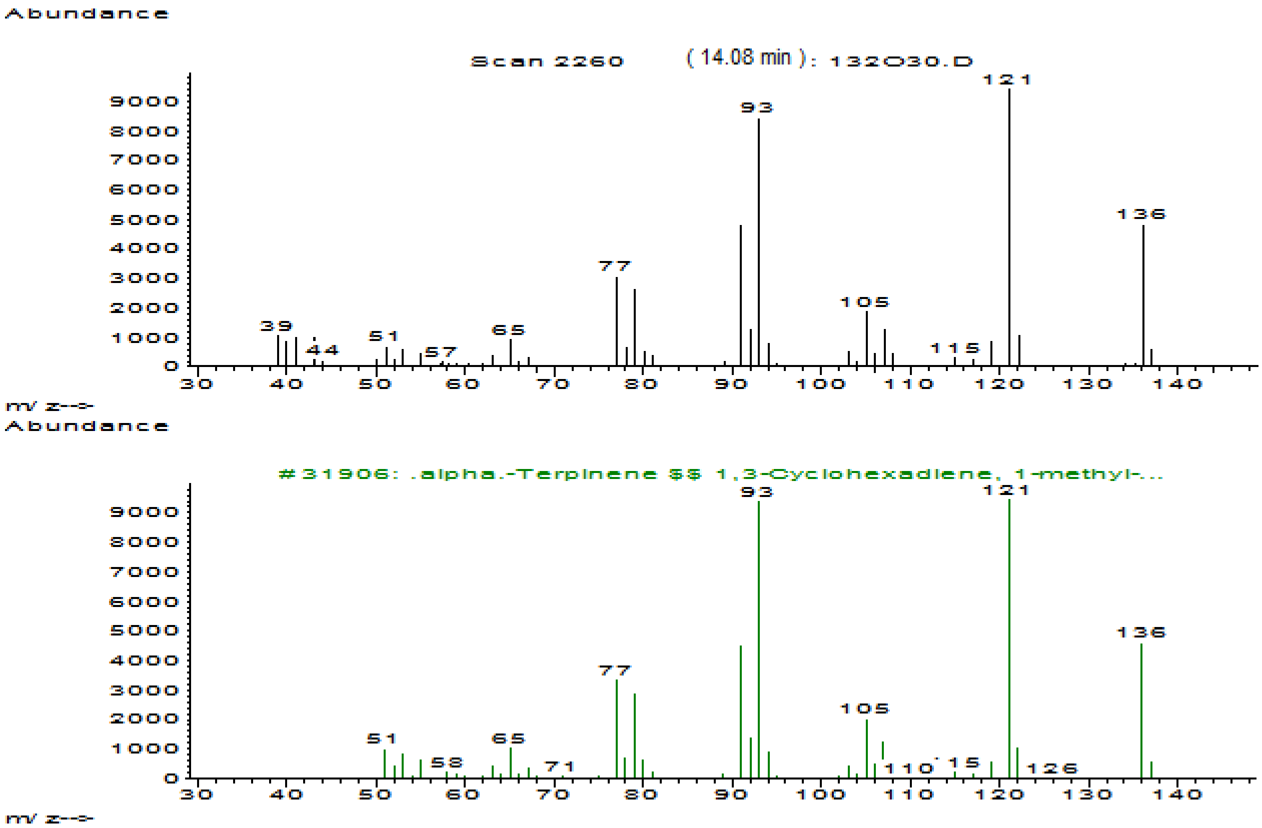

α-Terpinene

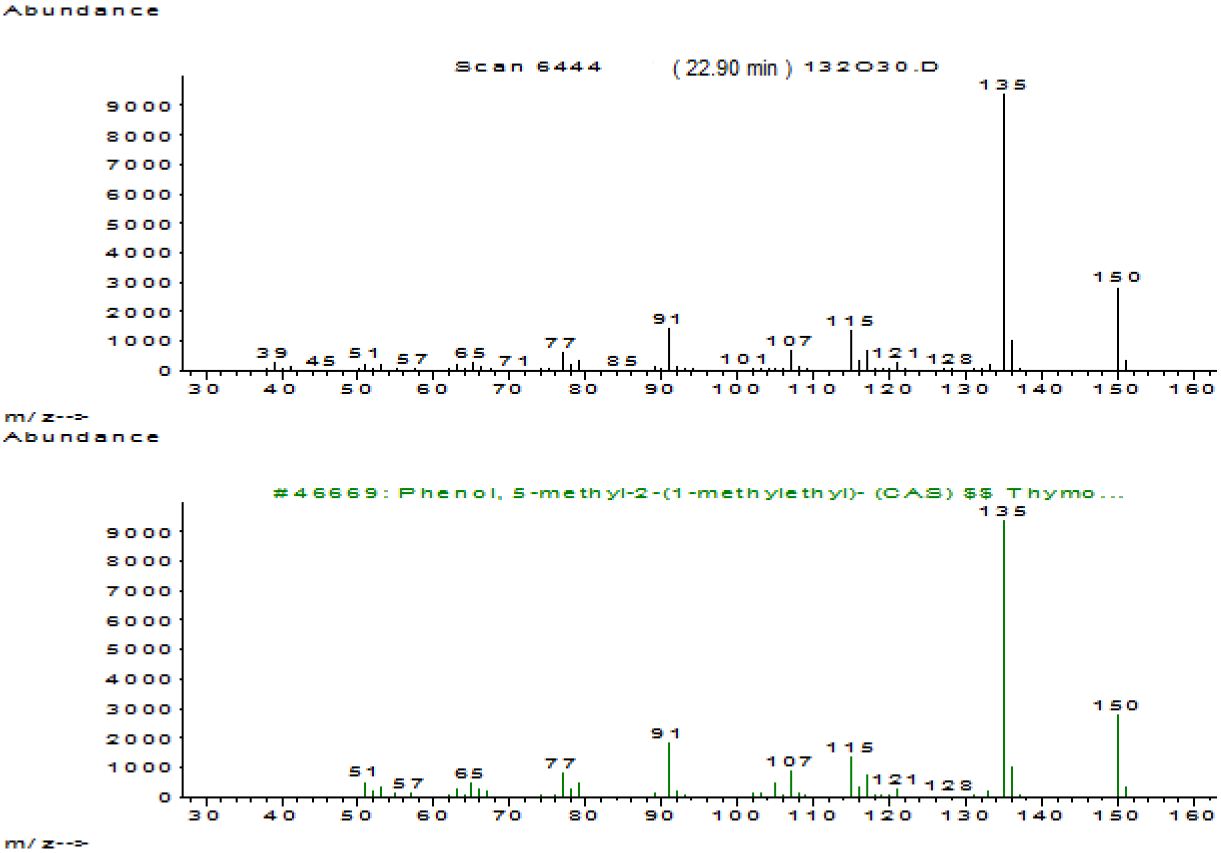

Thymol

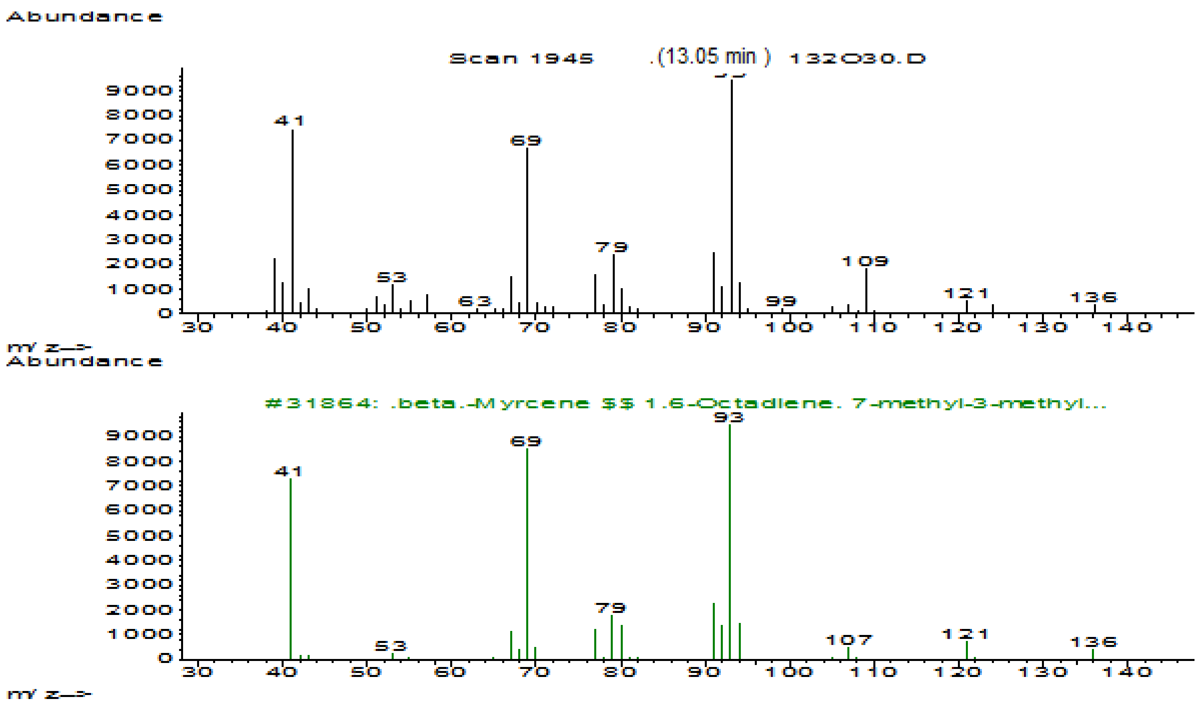

β-Myrcene

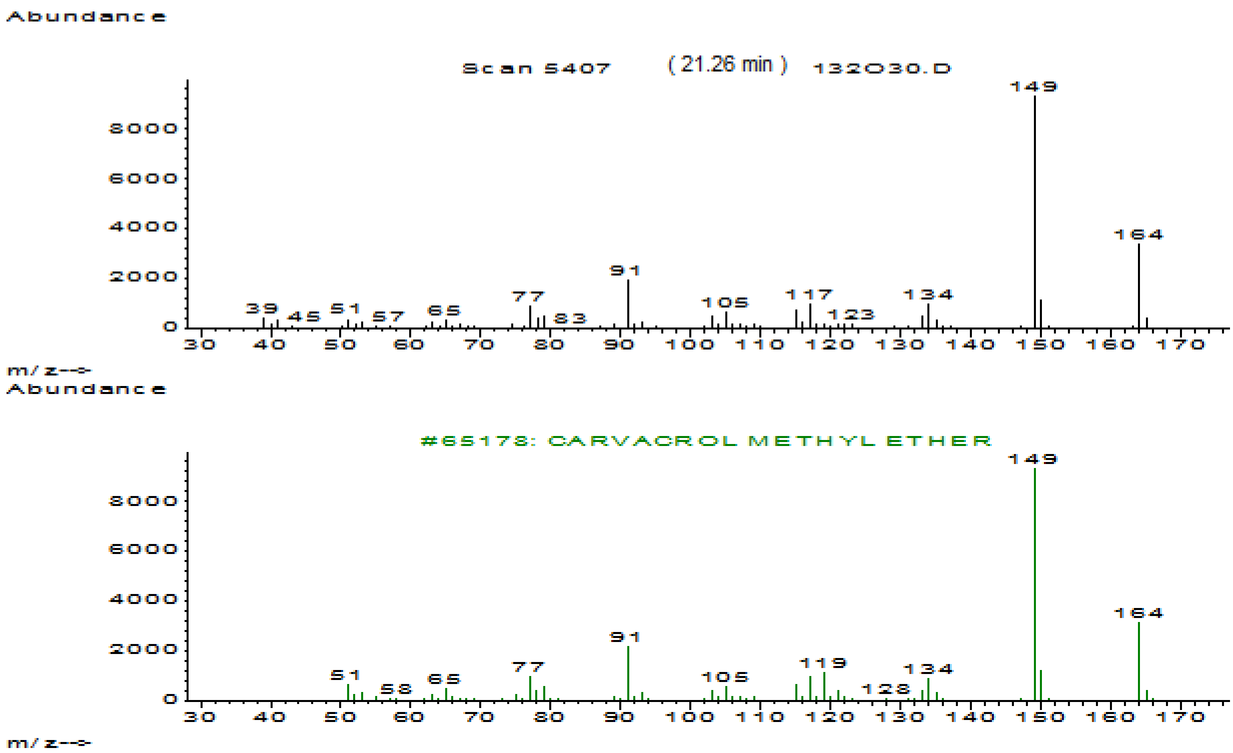

Carvacrol methyl ether

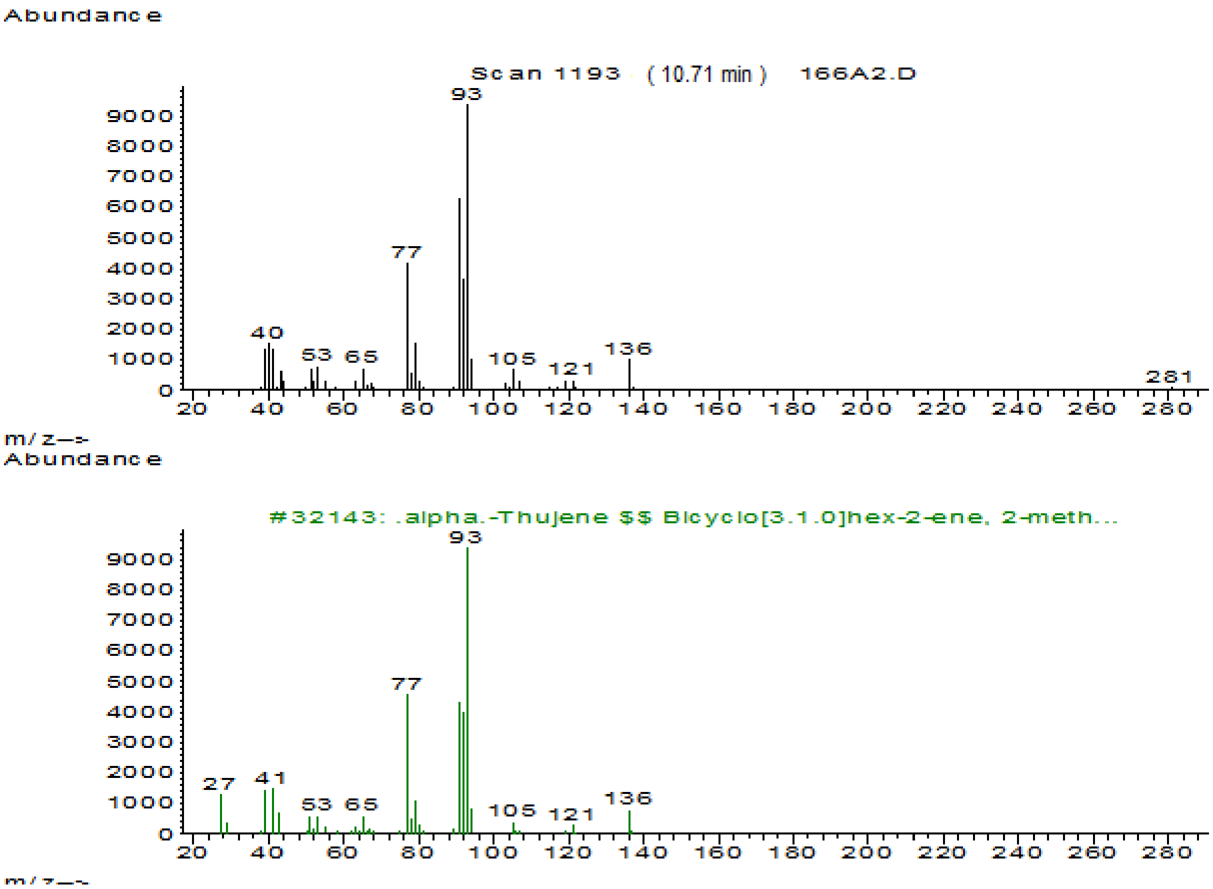
Abundance
Scan 1193 (10.71 min) 166A2.D
93
77
40
53 65
105
121
136
281
m/z-->
Abundance
#32143: .alpha.-Thujene $$ Bicyclo[3.1.0]hex-2-ene, 2-meth...
93
77
27
41
53 65
105
121
136
m/z-->

α-Thujene
